# Relationship of cardiorenal risk factors with albuminuria based on age, smoking, glycaemic status and BMI: a retrospective cohort study of the UK Biobank data

**DOI:** 10.1136/bmjph-2023-000172

**Published:** 2023-11-24

**Authors:** Debasish Kar, Aya El-Wazir, Mintu Nath, Penny Breeze, Karim Jetha, Mark Strong, Jim Chilcott, Melanie Jane Davies, Andrew Lee, Simon de Lusignan, Kamlesh Khunti, Amanda Adler, Elizabeth Goyder

**Affiliations:** 1 Nuffield Department of Primary Care Health Sciences, University of Oxford, Oxford, UK; 2 School of Health and Related Research, The University of Sheffield, Sheffield, UK; 3 Department of Infection, Immunity and Cardiovascular Disease, The University of Sheffield, Sheffield, UK; 4 Centre of Excellence in Molecular and Cellular Medicine, Suez Canal University, Ismailia, Egypt; 5 Medical Statistics Team, Institute of Applied Health Sciences, University of Aberdeen, Aberdeen, UK; 6 Dovercourt Surgery, Sheffield, UK; 7 Diabetes Research Centre, University of Leicester, Leicester, UK; 8 University Hospitals of Leicester NHS Trust, Leicester, UK; 9 Royal College of General Practitioners, London, UK; 10 Diabetes Trial Unit, University of Oxford, Oxford, UK

**Keywords:** Public Health, Preventive Medicine, Community Health

## Abstract

**Introduction:**

Smoking is harmful, and its cessation is recommended to prevent chronic kidney disease, which often begins with abnormal leakage of albumin in the urine, called albuminuria. Smoking cessation’s effect on albuminuria depends on the pack-years smoked, length of abstinence, body mass index (BMI) and glycosylated haemoglobin (HbA1c). Using the UK Biobank data, we examined the relationship between these cardiorenal variables and albuminuria.

**Methods:**

For this study, we selected a UK Biobank cohort with urinary albumin concentration (UAC) in the first and second visits. Participants were divided into progressor and regressor groups, where progressors were defined as those with increased UAC value, and regressors were those with decreased UAC value. Three different logistic regression models were fitted. In model 1, with a cohort design, we explored the impact of a change in age, HbA1c and BMI between the first and second visits and the UAC. In model 2 and 3, in a cross-sectional design, we explored which cardiorenal risk factors were associated with a rise or fall of UAC at the time point of the second visit. Results are expressed in OR and 95% CI.

**Results:**

The prevalence of albuminuria was highest in ex-smokers who started smoking between the ages of 13 and 18. With a mean duration of 51 months, there was no statistically significant relationship between smoking status and BMI with albuminuria. Each year of ageing and each unit of increase in HbA1c (mmol/mol) increased the odds of progression of albuminuria by 20% and 3%, respectively. In ex-smokers, at the time point of the second visit, each year of smoking increased, and each year of abstinence decreased the odds by 4% and 6%, respectively.

**Conclusion:**

Smokers should be supported to stop smoking and remain abstinent despite short-term weight gain. Childhood smoking should be actively discouraged.

**WHAT IS ALREADY KNOWN ON THIS TOPIC**

**WHAT THIS STUDY ADDS**

- Childhood smoking can increase the risk of albuminuria. Using the urinary albumin concentration at two time points in the UK Biobank study, participants showed that a rise in HbA1c after smoking cessation but not the body mass index is associated with the progression. In ex-smokers, at the time of the second visit, smoking increased the risk of albuminuria, and the duration of abstinence decreased the risk. Therefore, smokers should be supported to stop smoking despite concerns about potential weight gain. A holistic and multifactorial strategy should be adopted to prevent a rise in HbA1c after smoking cessation.

**HOW THIS STUDY MIGHT AFFECT RESEARCH, PRACTICE OR POLICY**

- This study can inform public health policy-makers to reinforce the message that smokers should quit and remain abstinent long term. As it is almost inevitable that smoking cessation is associated with weight gain, weight management should be an integral part of smoking cessation services. Irrespective of weight gain, smoking cessation can cause a rise in the HbA1c; therefore, close monitoring of HbA1c should also be part of smoking cessation services. Novel pharmacotherapy such as glucagon-like peptide 1 analogue with dual weight and HbA1c reduction action should be considered to help smokers quit and remain abstinent long term. Future research should explore the cost and effectiveness of this intervention to improve the successful quit rate and reduce the risk of vascular complications.

## Introduction

 Smoking and obesity are the leading preventable causes of mortality and morbidity worldwide. On average, the life expectancy of overweight smokers is 13 years lower than normal-weight non-smokers.^1^ People with cardiometabolic diseases are particularly at risk.^2 3^ To reduce the burden of complications arising from tobacco consumption in people with non-communicable diseases, the WHO set a target to reduce tobacco consumption from baseline in 2010 by 30% in its member countries by 2025.^4^ Due to raised awareness and public health intervention, the fourth WHO global tobacco trends report published in 2021 showed that tobacco consumption in adults had decreased considerably in its member countries. However, the report also highlighted that 38 million children aged 13–15 still smoke tobacco worldwide.^5^ Concurrently, due to rapid urbanisation in third-world countries and cheaper availability of high-calorie food in developed countries, childhood obesity is rising globally, increasing mortality and morbidity risk.^6 7^ Smoking and obesity are interlinked risk factors associated with microvascular complications such as retinopathy, neuropathy and nephropathy, and macrovascular complications such as coronary, cerebrovascular and peripheral artery disease.^8^,^11^ Therefore, the WHO has set a target of reducing childhood smoking to zero and reducing childhood obesity as its significant public health priority.^12^

The relationship between smoking, its cessation, obesity, risk of incident type 2 diabetes mellitus (T2DM) and vascular complications such as chronic kidney disease (CKD) is confusing and poorly researched. Compared with non-smokers, despite a lower body mass index (BMI), smokers have a higher risk of developing incident T2DM and CKD.^13 14^ Obesity, on the other hand, is an independent risk factor for T2DM and CKD.^15 16^ While smoking cessation is associated with a rise in cardioprotective high-density lipoprotein (HDL) cholesterol as early as 3 weeks after quitting,^17^ it is associated with a rise in glycosylated haemoglobin (HbA1c) and incident T2DM.^13 18^ While an increase in HDL cholesterol is likely to reduce the risk of vascular complications such as CKD, the impact of postcessation weight gain on renal function is unknown. Due to the rising number of people requiring renal replacement therapy (RRT) due to diabetic kidney disease,^19^ improving renal outcomes without increasing the risk of T2DM is a significant public health challenge.

Smoking is associated with insulin resistance and hyperinsulinaemia, which increases the risk of albuminuria.^20 21^ The risk is exceptionally high in young and adolescent obese smokers.^22^ To prevent children and adolescents from taking up the habit of smoking and quitting if they have already started smoking needs consistent and evidence-based advice. Most smokers start smoking in their early to late teens and continue in their adult lives.^23 24^ Key drivers for taking up smoking habits in this age group are to look slimmer and manage stress, leading to an obesity paradox and a lifelong reliance on nicotine as a stress reliever.^25 26^ Consequently, young and adolescent smokers enter adulthood with a disproportionately higher risk of T2DM and microvascular and macrovascular complications.

Smoking cessation, on the other hand, improves insulin sensitivity^27^ but paradoxically increases the risk of incident T2DM,^28^ which can deter smokers from quitting. A recent US study showed that the significant barriers for young and adolescent smokers to quit and remain abstinent are post-cessation increases in stress, anxiety, BMI and nicotine withdrawal symptoms. On the other hand, the major facilitator to successful quitting is improved physical fitness and the higher cost of tobacco.^29^ In addition, smokers from deprived socioeconomic backgrounds find postcessation weight gain a significant trigger for resuming smoking after a brief period of abstinence.^30^ Stopping and starting smoking instead of sustained abstinence is counterproductive and may not reduce the risk of vascular complications such as CKD.^31 32^

Albuminuria is an important hallmark for both microvascular and macrovascular complications.^33 34^ Smoking is strongly associated with albuminuria, and its cessation attenuates the risk.^35^ However, the research evidence of the effect of postcessation weight gain on vascular complications is inconsistent. Some studies reported that microvascular and macrovascular risk could be reduced after smoking cessation if postcessation weight gain can be prevented.^36 37^ In contrast, some other studies contradicted this observation and suggested that those who had postcessation weight gain had a lower risk of a major adverse cardiovascular event and mortality than those who lost weight.^38^ Therefore, the anxiety of further weight gain, risk of developing T2DM, worsening of glycaemic control and unknown impact on vascular outcomes are the major deterrents for obese smokers to quit.^39^ Although public health guidelines are unambiguous about the harm of smoking and the benefit of smoking cessation, they are less clear about the cardiometabolic consequences of smoking cessation and how to manage them.

This study explored how the differences in age, BMI, systolic blood pressure (SBP), HbA1c, creatinine, HDL and total cholesterol (TC) values between the first and second visits impact albuminuria progression based on smoking status and gender. In a subgroup analysis of ex-smokers, we examined the effect of male sex, age, diastolic blood pressure (DBP), waist circumference, cholesterol, HbA1c, years of smoking before quitting and years of abstinence on the odds of albuminuria at the time point of the second visit.

## Materials and methods

### Study cohort and design

**Figure 1:**
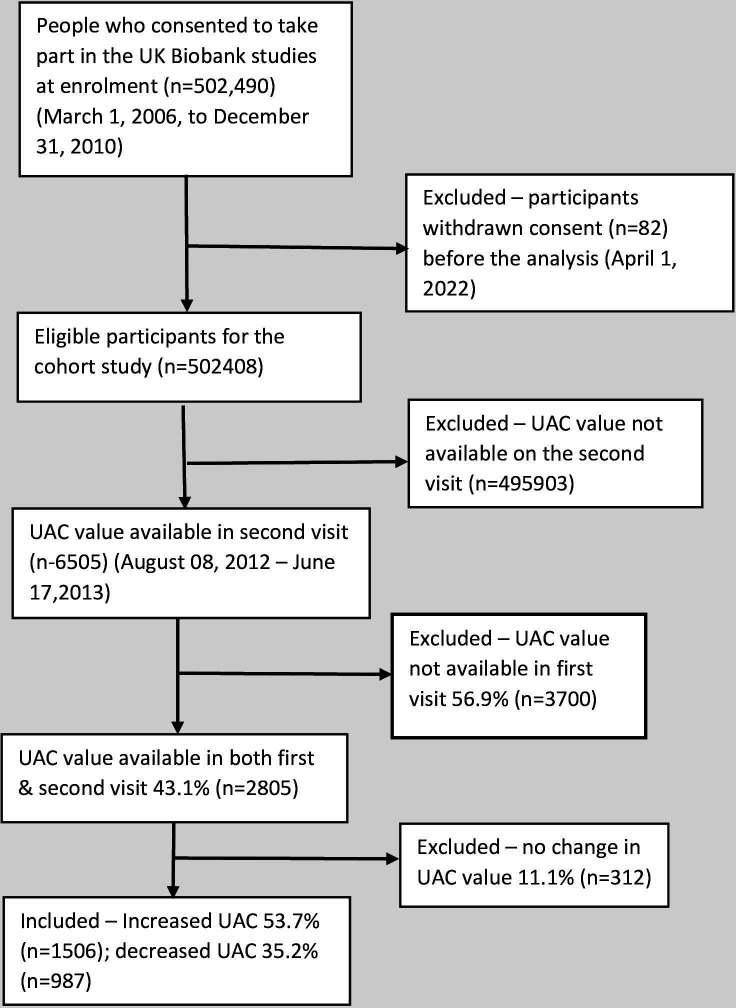
Flow chart of selection of study population. UAC, urinary albumin concentration.

We conducted a retrospective cohort study on volunteers of UK Biobank data who had urinary albumin concentration (UAC) values in both the first and second visits. The age range of volunteers on the first visit was 40–70 years, and on the second visit was 44–76 years. The initial visit was between 13 March 2006 and 01 October 2010, and the follow-up was between 8 August 2012 and 7 June 2013. Out of a total of 502 490 participants, 30.42% (n=1 52 864) provided urine samples on the first visit, and from a total of 20 346 participants, 31.97% (n=6505) provided urine samples on the second visit. Out of them, the corresponding UAC value was available in the first visit in 43.1% (n=2805); between the visits, the UAC value was unchanged in 11.1% (n=312), increased in 53.7% (n=1506) and decreased in 35.2% (n=982) (figure 1). The data field description in the UK Biobank was ‘microalbumin in urine’.

### Selection process and data collection

In the follow-up visit, the UK Biobank participants (n=20 346) completed a questionnaire about demographic details, smoking status and medical history. They also had physical examinations to measure height, weight and blood pressure by a qualified research nurse. Those who agreed to provide blood, urine and saliva samples were also analysed. Data for age, sex, the age at which smokers started and stopped smoking, the number of years they smoked, and when diabetes and hypertension were diagnosed were obtained from the questionnaire. Duration of smoking was calculated by subtracting the age they started smoking from the age they stopped smoking; duration of abstinence was calculated by subtracting the current age on the second visit and the age they stopped smoking. All the data were verified by the lead author (DK) and another study team member (AE-W). The data were reported using Strengthening the Reporting of Observational Study reporting guidelines for retrospective studies.

### Patient and public involvement

In this project, patients and the public were not directly involved.

### Inclusion and exclusion criteria

The inclusion criteria were the availability of UAC values at two time points, visit 1 and visit 2. Those who did not have the UAC value on both the first and second visits were excluded. Adults aged 18 years or over were included, and those aged below 18 years were excluded. UK Biobank published a list of participants who withdrew their consent to take part in the study. We removed their data from the analyses. Smoking data were obtained from the questionnaire the participants filled out. Those participants who did not want to disclose their smoking status were classed as missing data and were not used for statistical analyses.

### Exposure and covariates

The population of interest was those who had UAC values in both the first and the second visit. Based on the UAC values between the visits, the population was divided into three groups—progressors, whose UAC value increased between the first and second visits; regressors, whose UAC value decreased in the second visit; and unchanged, whose UAC values were the same in both the first and the second visits. Smokers were classed as active smokers (smoked cigarettes excluding electronic cigarettes for 12 months or longer) at the second visit, non-smokers were those who never smoked and ex-smokers were those participants who smoked in the past but had given up at the time of the second visit and were abstinent for at least a year. Covariates were age, sex, BMI, waist circumference, age started and stopped smoking, serum creatinine, HbA1c, blood pressure and cholesterol at the second visit.

A Kolmogorov-Smirnov test showed that the distribution of all the numerical variables was parametric except age and UAC in visit 2 (online supplemental material). These two variables are expressed in median and range. All other numerical values are expressed in mean (SD). BMI was expressed in kg/m^2^; SBP and DBP were expressed in mm of Hg; cholesterol, HDL and low-density lipoprotein (LDL) were expressed in mmol/L, and HbA1c was expressed in mmol/mol and %. This study did not include smoking e-cigarettes or consuming tobacco in any other form except smoking cigarettes.

### Outcome variables

For this study, we used the Kidney Disease Improving Global Outcomes (KDIGO) guideline, which defined normoalbuminuria as UAC value in a spot sample of urine <20 mg/L, microalbuminuria as 20–200 mg/L and macroalbuminuria as >200 mg/L.^40^ Albuminuria was defined as any value above>20 mg/L, and an increase in the UAC value was ≥1 mg/L, the decrease was ≥−1 mg/L, and no change was the same value in the first and second visits. However, this change in UAC values in two visits is not clinically significant. The study’s primary outcome was a change in the UAC values between the first and second visits. The secondary outcomes were changes in the HbA1c, blood pressure, serum creatinine, TC, HDL, LDL and BMI between the two visits.

### Software used

All the analyses were carried out using IBM SPSS V.28.0.1.1 and R V.4.2.2.

### Statistical analyses

The primary analysis was a cohort study conducted on the study population who had the UAC values in the first and the second visits. In this part of the analysis, a logistic regression model was fitted in a cohort design with differences in numerical cardiorenal risk factors of age, BMI, SBP, HbA1c, creatinine, HDL and TC between the first and second visits. In the same model, the differences in the odds of albuminuria in males versus females, ex-smokers versus non-smokers, and current smokers versus non-smokers were elucidated. In a subgroup analysis, using a cross-sectional design, a logistic regression model was fitted in ex-smokers to examine the relationship of the male sex, age, DBP, waist circumference, cholesterol, HbA1c, duration of smoking and duration of abstinence with an increase and decrease in UAC value at a time point of second visit. Participants who did not have their UAC value on both the first and second visits and those who did not have any change in the UAC were classed as missing values. Missing values were excluded from the analysis, as this study focuses on exploring the relationship between cardiorenal risk factors and change in the UAC value over a two-time period. However, a separate analysis was conducted on missing values to examine if the missing values were distributed randomly (online supplemental material).

A descriptive analysis was carried out to summarise the cardiorenal characteristics of the study participants. A χ^2^ test was conducted to explore the significance of cardiorenal risk factors based on progression, smoking and glycaemic status. Finally, a Student’s t-test examined the statistical significance (<0.05) of the difference in numerical variables in the progressor and regressor groups.

Three separate logistic regression models were fitted using sex and smoking status as binary variables and a change in age, BMI, SBP, HbA1c, creatinine, HDL and cholesterol between the first and second visits as numerical variables. In model 1, the model was fitted on the progressor and regressor groups to evaluate the association of cardiorenal risk factors with changes in albuminuria between the first and second visits. Further logistic regression was conducted on the subset of ex-smokers. Explanatory variables in subset analyses were in model 2a, the duration of smoking and waist circumference; in model 2b, the duration of smoking and BMI; and in model 2c, the duration of smoking without BMI and waist circumference. Similarly, the explanatory variables were in model 3a, the duration of abstinence and waist circumference; in model 3b, the duration of abstinence and BMI; and in model 3c, the duration of abstinence without BMI and waist circumference.

## Results

After excluding 82 participants who withdrew their consent, we included 502 408 participants for this study. Based on the UAC value in the second visit, 6505 participants were selected for descriptive analysis, and their baseline characteristics are summarised in table 1. Among the included participants, 46.1% (n=2999) were female, and 53.9% (n=3506) were male. Based on the HbA1c values in the second visit, 60.8% (n=3954) had normoglycaemia, 4.9% (n=318) had HbA1c in the pre-diabetes range and 4.4% (n=288) had at the diabetes range. 29.9% (n=1945) had no HbA1c value at the second visit. 43.1% (n=2805) had UAC values available on both the first and the second visit. Of them, 23.2% (n=1506) were progressors, 15.2% (n=987) were regressors and 4.8% (n=312) did not have any change. People who did not have UAC values in both the first and the second visits and those whose UAC value was unchanged were classed as missing values. Analyses of the missing values showed that they were distributed randomly, with no systematic bias (online supplemental material)(online supplemental material).

**Table 1:** Baseline characteristics of eligible study participants in the second visit

Variable	Mean/median	SD/IQR	No (n)
Age (years) (median)	64	11	6505
Waist circumference (cm)	93.1	13.8	6497
Age started smoking (years) (ex-smokers)*	17.2	3.6	532
Years smoked before quitting (ex-smokers)*	22	12.1	532
Age stopped smoking (years)*	39.8	12.7	532
Years of abstinence†	23.4	13.5	532
Age diabetes diagnosed**	52.2	12.2	386
Age hypertension diagnosed (years)*	47.0	16.4	1979
Systolic blood pressure (mm Hg)	138	18.8	6081
Diastolic blood pressure (mm Hg)	83	10.2	6081
Body mass index (BMI) (kg/m^2^)	27.7	5	6485
Urinary albumin concentration (mg/L)	27.65	103	6505
Serum creatinine (µg/dL)	77.3	16	462
HbA1c (mmol/mol, %)	37.4 (5.6)	7	4560
Total cholesterol (mmol/l)	5.6	1.2	5712

BMI – —Nnormal weight (20–25 kg/m2), overweight (25–30 kg/m2), obese (>30 kg/m2).

* Obtained from the questionnaire, not verified.

† Worked out from the date of the second visit and the date of quitting.

HbA1cglycosylated haemoglobin

On the second visit, the mean age of study participants was 62.8±7.5 years, the mean age of starting smoking in ex-smokers was 17.2±3.6 years, the mean age of starting smoking in current smokers was 18.4±7.2 years, the mean age of stopping smoking was 39.8±12.7 years. The mean length of smoking before quitting was 22.3±11.8 years, and the mean length of smoking in current smokers was 39.2±9.4 years. The mean age of diabetes diagnosis was 52.8±12.7 and the mean BMI was 28.7±5.0 (table 1).

A χ^2^ test was conducted based on progression, smoking and glycaemic status. While diabetes status at the second visit (pre-diabetes and diabetes) was statistically significantly associated with the progression of UAC value, smoking status was not. An independent sample Student’s t-test showed that there was a statistically significant relationship between progression status age in visit 2, creatinine and DBP; there was no statistically significant relationship with SBP, BMI and HbA1c (online supplemental materials). A Spearman’s ranked correlation analysis showed evidence of a statistically significant relationship between UAC value in the second visit with age, SBP and DBP, waist circumference, BMI, TC, serum creatinine, HbA1c, HDL and LDL, duration of smoking of current and ex-smokers (online supplemental material).

**Figure 2:**
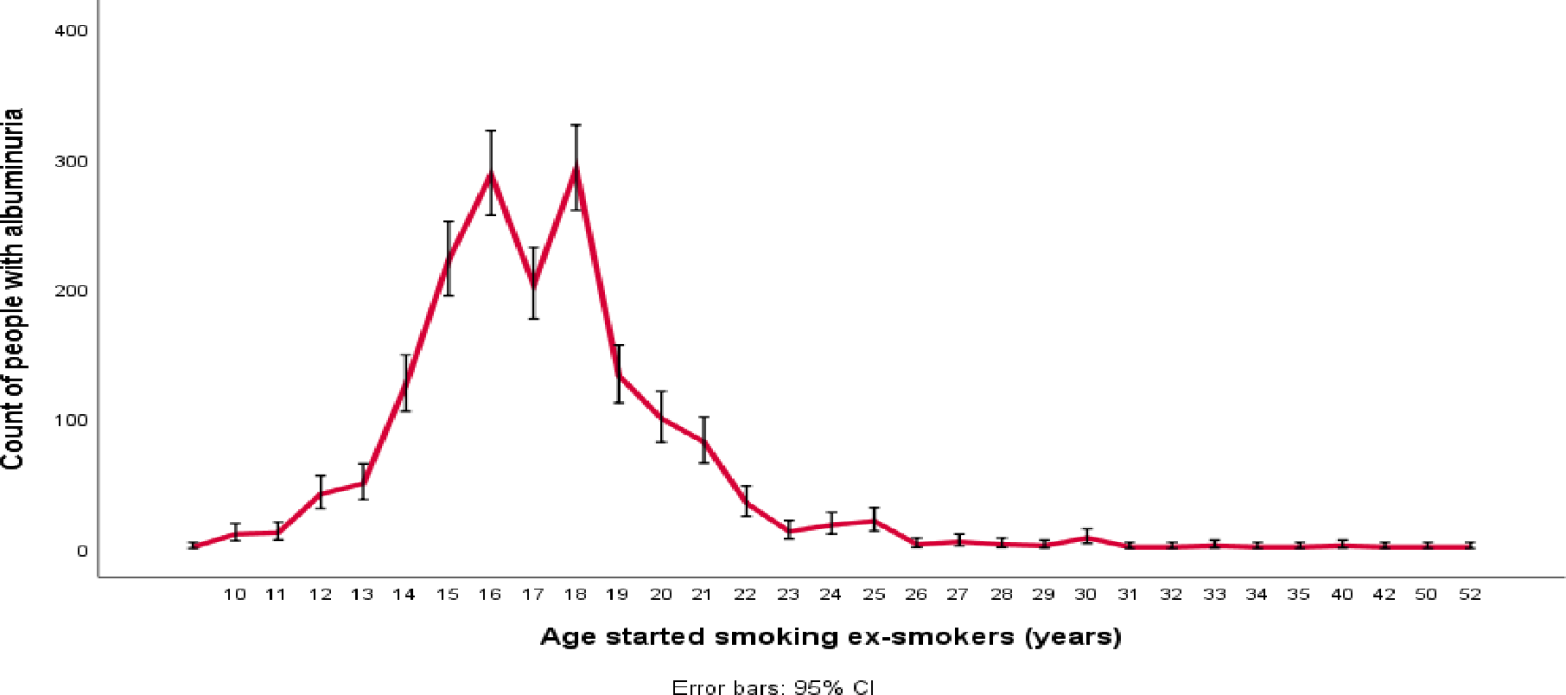
Prevalence of albuminuria based on the age started smoking.

**Figure 3:**
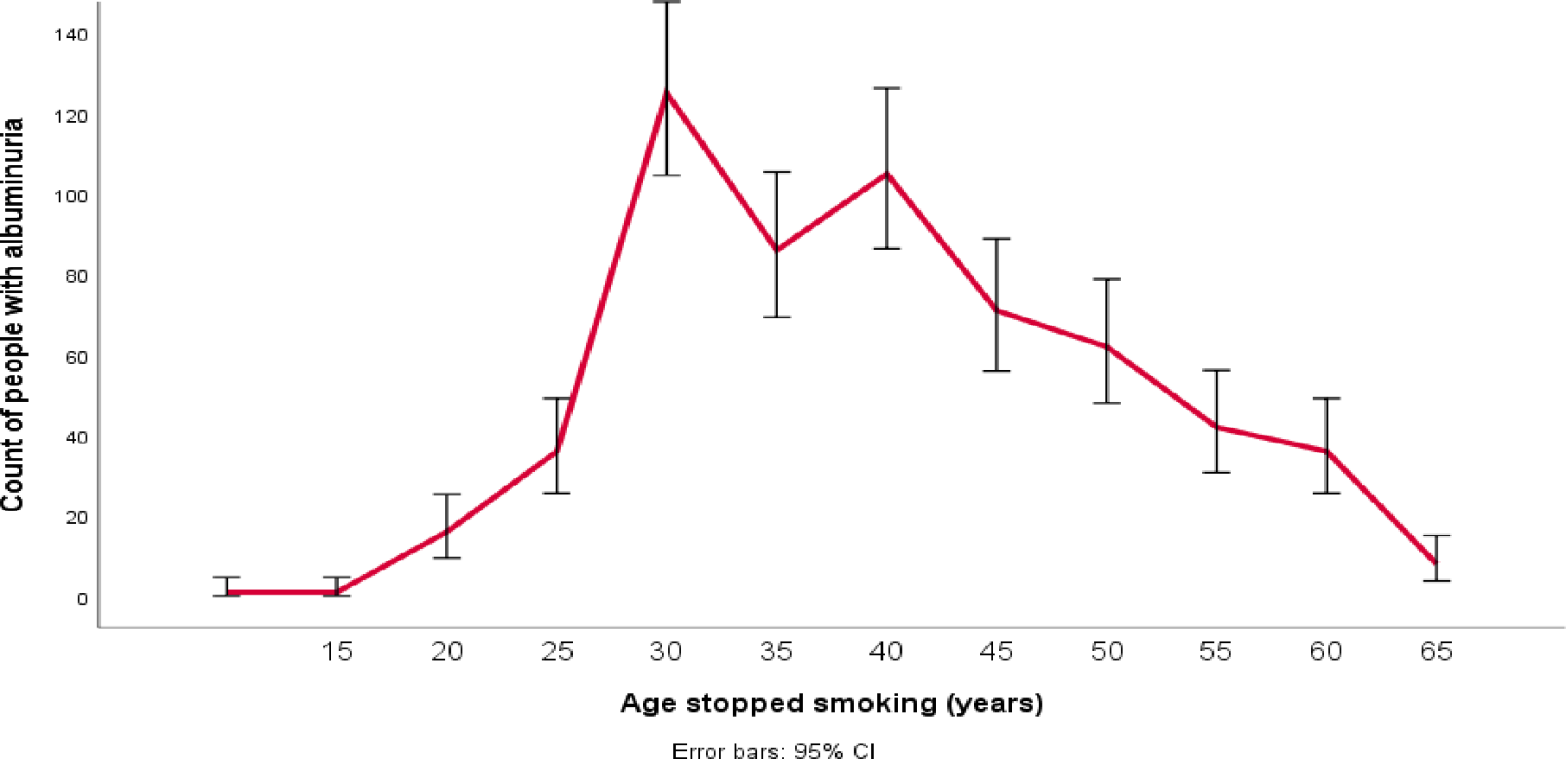
Prevalence of albuminuria based on the age at which stopped smoking.

The number of ex-smokers whose UAC value increased in the second visit was plotted against the age when they started and stopped smoking. The plot showed that the prevalence of albuminuria was higher among those ex-smokers who started smoking between the ages of 13 and 18 (figure 2) and those who stopped smoking between the ages of 15 and 35 (figure 3).

A logistic regression model was fitted in model 1 to explore the relationship between the progression and regression of albuminuria between the two visits and cardiorenal risk factors of age, sex, smoking status, BMI, SBP, HbA1c, creatinine, HDL and TC. Changes in age and HbA1c had a statistically significant association with the progression and regression of albuminuria. None of the other covariates had any statistically significant association with the progression or regression of albuminuria (table 2).

**Table 2:** Model 1µfactors predicting the progression/regression of albuminuria (n=2493)

Variables	OR	P value	95% CI	Effect size (SE)
Male versus female	1.22	0.14	0.94 to 1.58	0.20 (0.13)
Age (visit 2–visit 1)	1.20	<0.01	1.06 to 1.36	0.18 (0.06)
BMI (visit 2–visit 1)	1.03	0.48	0.95 to 1.10	0.03 (0.04)
SBP (visit 2–visit 1)	1.0	0.88	0.99 to 1.01	0.001 (0.007)
HbA1c (visit 2–visit 1)	1.03	0.02	1.00 to 1.05	0.03 (0.01)
Creatinine (visit 2–visit 1)	0.99	0.15	0.98 to 1.00	−0.008 (0.006)
HDL (visit 2–visit 1)	0.53	0.05	0.28 to 1.01	−0.64 (0.33)
Total cholesterol (visit 2–visit 1)	1.08	0.21	0.95 to 1.23	0.08 (0.06)
Ex-smoker versus non-smoker (visit 2)	1.22	0.50	0.69 to 2.15	0.20 (0.29)
Current smoker versus non-smoker (visit 2)	0.72	0.52	0.26 to 1.97	−0.33

BMIbody mass indexHbA1cglycosylated haemoglobinHDLhigh-density lipoproteinSBPsystolic blood pressure

### Subset analyses on ex-smokers

From 2805 people with the UAC values available on both the first and the second visits, ex-smokers who had information on their current smoking status and the date of starting and stopping smoking were selected. Nineteen per cent (n=532) of the selected participants had the above data. Among them, 35.3% (n=188) were female, and 64.7% (n=344) were male. The mean age was 63.4±6.9 years, the mean duration of smoking before quitting was 21.3±11.3 years, the mean age of starting smoking was 17.2±3.3 years, the mean age of stopping smoking was 38.5±11.1 years, and the mean duration of abstinence was 24.9±11.7 years.

**Figure 4:**
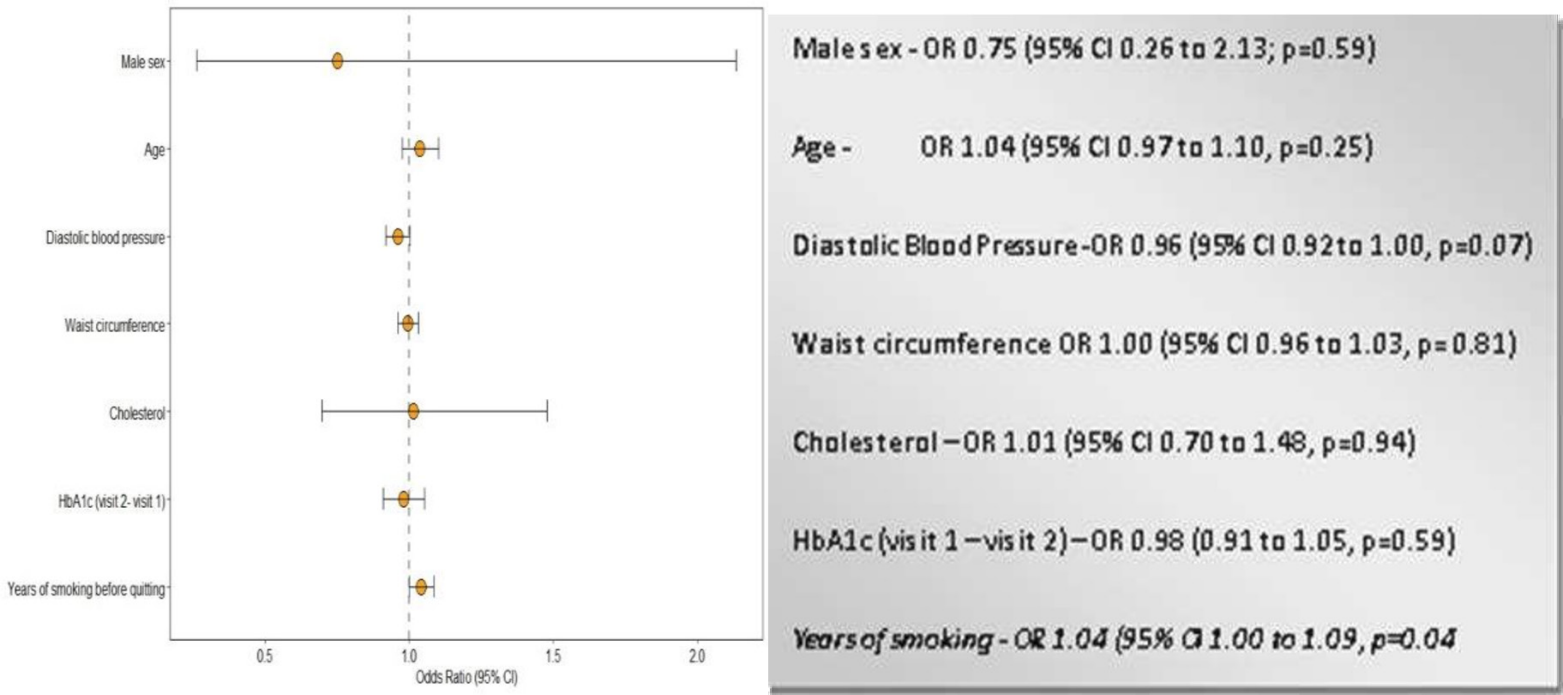
Predictors of albuminuria based on the years of smoking. HbA1c, glycosylated haemoglobin.

In model 2a, the duration of smoking was fitted with age, sex, waist circumference, cholesterol, DBP and change in HbA1c between the first and second visit. None but the duration of smoking before quitting was statistically significantly associated with the progression of albuminuria (figure 4). However, in model 2b, when BMI replaced waist circumference, none of the above cardiorenal risk factors had any statistically significant relationship with the progression of albuminuria. Finally, in model 2c, when both BMI and waist circumference were removed from the model, the duration of smoking remained a statistically significant predictor of albuminuria in ex-smokers, suggesting that the duration of smoking before quitting is an independent determinant of the progression of albuminuria (online supplemental material).

**Figure 5:**
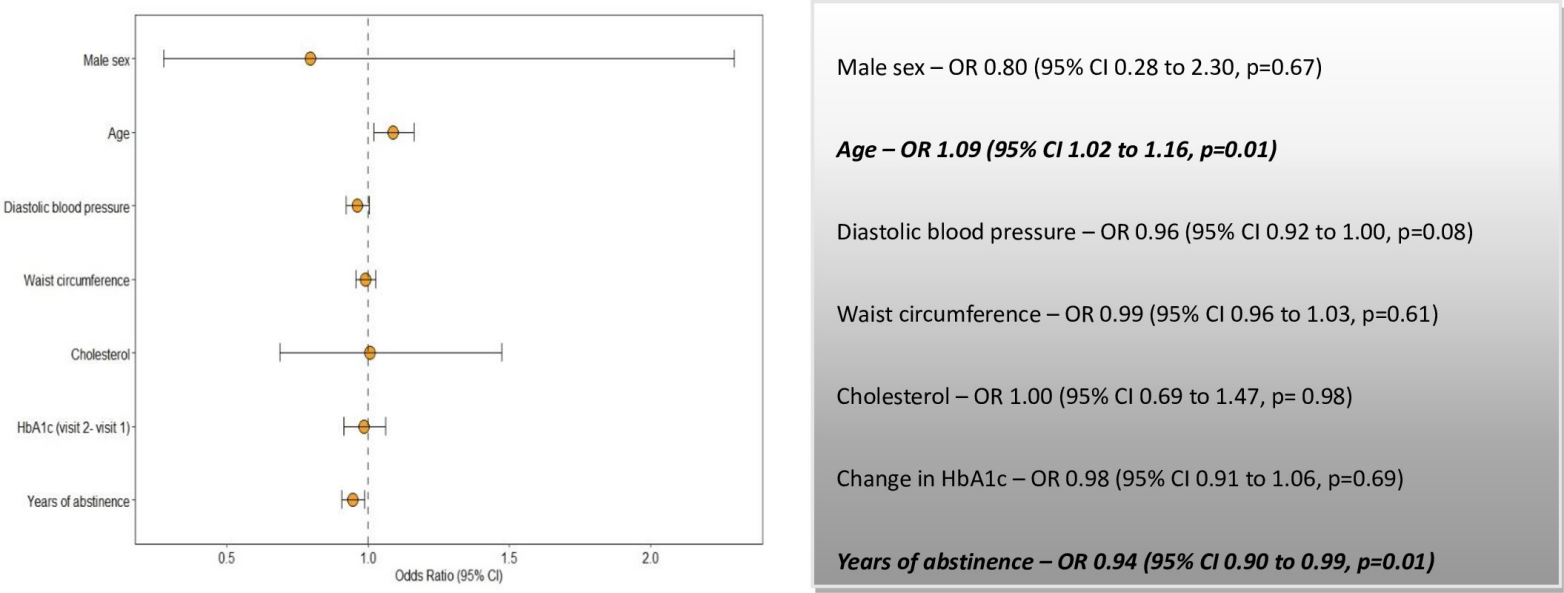
Predictors of albuminuria based on years of abstinence. HbA1c, glycosylated haemoglobin.

In model 3a, the duration of abstinence was fitted with age, sex, DBP, cholesterol, waist circumference and the change in HbA1c between the first and the second visit. Apart from the age in the second visit and the duration of abstinence, no other confounding variables had any statistically significant relationship with the progression of albuminuria (figure 5). In model 3b, waist circumference was replaced with BMI; in model 3c, neither BMI nor waist circumference was used. Age and the duration of abstinence remained statistically significant predictors of progression in both models, suggesting that the longer the ex-smokers remained abstinent, their risk of progression declined, irrespective of change in BMI and waist circumference (online supplemental material).

## Discussion

In this study, at the time point of the second visit, the number of people with albuminuria was significantly higher in ex-smokers who started smoking between the ages of 13 and 18 years and stopped smoking between the ages of 25 and 35 years. After adjusting for confounding variables of age, male sex, DBP, waist circumference, cholesterol and HbA1c, in ex-smokers, each year of smoking before quitting increased the odds of albuminuria by 4%, each year of ageing increased the odds by 9%, and each year of abstinence reduced the odds by 6%.

This study showed that with a mean duration of 51.7±10.9 months between the first and second visits, ageing and change in HbA1c were the only statistically significant predictors of the progression of albuminuria. Each year of ageing increased the odds of progression by 20%, and each unit of HbA1c (mmol/mol) increased the odds by 3%. Between the two visits, changes in smoking status, BMI, waist circumference, SBP, cholesterol, creatinine and HDL had no statistically significant association with the progression of albuminuria, suggesting that short-term changes in those risk factors may not impact the progression.

Active and passive exposure to cigarette smoking at an early stage of life may increase the risk of CKD and predispose to faster disease progression requiring RRT.^14 41^ Previous studies have shown that young and adolescent smokers are at a higher risk of developing microvascular and macrovascular complications, including a rapid decline in the estimated glomerular filtration rate (eGFR) and developing hypertension.^42^,^44^ The finding of this study is in keeping with existing knowledge.

Smoking cessation is almost inevitably associated with weight gain due to a reduction in basal metabolic rate, increased appetite and craving for high-calorie food to mitigate nicotine withdrawal symptoms.^45^,^47^ Nicotine and other toxic chemicals in cigarettes destroy the structure and function of insulin-secreting pancreatic beta-cells of Islets of Langerhans and damage glomerular vascular endothelium, predisposing to CKD.^48^,^50^ Therefore, smoking cessation is recommended despite postcessation weight gain.^51^ This is supported by our study, which showed that BMI and waist circumference following smoking cessation did not have a statistically significant effect on the progression of albuminuria.

The study also showed that the longer the duration of smoking, the higher the risk of albuminuria in ex-smokers. Prolonged exposure to nicotine and other toxic chemicals in cigarettes may damage the renovascular structure and function irreversibly, which may not recover despite smoking cessation. When the eGFR starts dropping, smoking cessation may not reverse the function back to normal. Younger smokers are particularly at risk, as nicotine exposure at this age can interfere with the development of renovascular structure, which may not be reversible despite smoking cessation at a later stage of life.^52^

In this study, we have demonstrated that the longer the duration of abstinence, the lower the risk of progression of albuminuria. Previous studies support this finding of this study. The STENO-2 follow-up cohort study showed that a multifactorial holistic intervention, including a total abstinence from smoking, could prevent a decline in renal function and reduce the risk of cardiovascular mortality 21 years after the intervention.^53^

The finding of this study that a rise in HbA1c between the first and the second visits was an independent predictor of albuminuria is supported by previous studies.^54 55^ Young obese smokers are particularly at risk. During puberty, there is a physiological decline in insulin sensitivity by almost 50% due to the action of growth hormone.^56^ To compensate, pancreatic β-cell must increase their insulin secretion by 50%,^57^ but in smokers, this compensatory pathway becomes dysfunctional.^58^ Nicotine and other toxic chemicals in cigarettes cause premature apoptosis of functioning β-cell of the Islets of Langerhans.^48^ Therefore, lifestyle intervention and pharmacotherapy should be offered to retain functioning β-cell. Glucagon-like-peptide-1 analogues such as Liraglutide and Semaglutide have shown promising results in reducing HbA1c, losing weight and preventing premature apoptosis of the β-cell.^59 60^ Likewise, SGLT-2 inhibitors are effective in preventing the progression of CKD and heart failure and are licensed in the UK for use in people without T2DM for CKD and heart failure.^61^,^64^ However, they are not tried in obese smokers to quit and remain abstinent. This should be a priority area for future research.

In this study, smoking status did not significantly impact the UAC values between the first and second visits. A 51-month duration was not long enough to observe any significant changes in albuminuria. In the KoreanN cohort study for Outcomes in patients With CKD (KNOW-CKD) prospective observational cohort study, the researchers analysed data for 1951 patients enrolled in the KNOW-CKD from 2011 to 2016 based on smoking behaviour. The study’s primary outcome was a composite of reduction ≥50% in eGFR, initiation of dialysis or renal transplantation. After a mean follow-up of 3 years, the HRs (95% CI) of primary outcome were 1.09 (0.73 to 1.63), 1.48 (1.00 to 2.18) and 1.94 (1.35 to 2.77) in smokers who smoked <15, 15–29 and ≥30 cigarettes/day, respectively. Similarly, after smoking cessation, the progression of kidney disease was attenuated. The adjusted model, including the decline in eGFR and log-transformed urine protein-to-creatinine ratio, showed that after smoking cessation, compared with non-smokers, the risk of progression of CKD in ex-smokers with<10, 10–19 and ≥20 years of abstinence were 1.84 (1.28 to 2.66; p=0.001), 1.44 (0.85 to 2.42; p=0.176) and 1.35 (0.80 to 2.28, p=0.267), respectively.^65^ The risk of progression of CKD remained high in all three groups, but except for the <10 years group, the HR did not reach the level of statistical significance. Further research is needed to elucidate how to reduce the risk of progression of CKD in ex-smokers.

The epidemiology of renovascular complications has changed exponentially over the last 20 years, and therefore, current practice and policy need to be carefully evaluated. Existing global diabetes management guidelines and prevention of complication models are heavily dependent on the UK Prospective Diabetes Study (UKPDS).^66^,^68^ In the UKPDS study participants, the prevalence of albuminuria 10 years after the diagnosis of T2DM was approximately 24.9%, which is no longer the case.^69 70^ Diabetes UK 2019 report showed that one- third of people had already developed one or more microvascular complications at the onset of T2DM.^70^ Despite adhering to the National Institute for Health and Clinical Excellence guidelines developed from the UKPDS model and high attainment score in the Quality Outcome Framework for HbA1c and blood pressure, the Renal Registry UK report 2022 suggests that the number of new people registered for RRT in 2020 was 7323, which was identical to previous years. The report suggests that the actual number who qualified for RRT could be higher as many people refused to have RRT during the coronavirus pandemic. The proportion of people requiring RRT due to diabetes had increased from 24.1% in 2011 to 30.5% in 2020. Among those who needed RRT due to diabetes, 31% were between the ages of 45 and 54, and 40% were between the ages of 55 and 64 years, which is considerably lower than the UKPDS study participants.^71^

In contrast to the UKPDS study participants, where the mean age of diagnosis of T2DM was 51 years, T2DM is no longer an exclusive disease of older and middle-aged people. Between 2007 and 2015, in the UK, the incidence of T2DM in people aged 17 years or less increased from 0.53 to 0.72 cases per 100 000 person-years.^72^ Likewise, vascular complications develop at any stage of metabolic deregulation, even at the stage of pre-diabetes. In our recent cross-sectional study, we have demonstrated that almost 35% of smokers with pre-diabetes had already developed albuminuria.^73^ A recent cohort study reported that between 2009 and 2018, in the UK, the incidence rate of T2DM declined by a third, compared with its peak in 2013–2014, but the incidence of pre-diabetes had tripled.^74^ In 2022, one in three adults in the UK had pre-diabetes.^75^ Therefore, to prevent the rising surge of younger people requiring RRT, young and adolescent smokers should be a priority group and should be offered support to quit, irrespective of their diabetes status.

After stopping smoking, remaining abstinent is challenging and postcessation weight gain is a major barrier. An average smoker makes approximately 16 attempts before successfully quitting.^76^ Multiple studies have shown that the relapse rate is significantly higher in those quitters who gained weight beyond 3 months after quitting.^77 78^ Therefore, a weight management programme and careful monitoring of glycaemic control should be offered as a package in the smoking cessation programme.

### Strengths and limitations

To our knowledge, this is the first study to explore the impact of smoking cessation on the progression of albuminuria in the UK. A study in the Korean population showed a graded reduction in the risk of kidney disease following smoking cessation. This study has identified an area of knowledge gap, and a bigger study on real-world data can be conducted to guide national policy on smoking and kidney disease.

However, there are several weaknesses in the study. Although the study was intended to explore the progression of albuminuria over a period of time, there were data limitations. A 51-month duration is not a long enough period to observe any meaningful change in the progression or regression of albuminuria. The UAC value for progression and regression was ±1 mg between the two visits, which is not clinically significant, and it is within the margin of SD. To understand the actual impact of smoking and its cessation on the progression of albuminuria real-world data is needed, rather than the data from volunteers over a longer period of time.

Smoking status was determined from the questionnaire and was not verified. Similarly, the age of starting and stopping smoking was also patient reported and open to recall bias. UAC was used as the parameter for progression and regression, although the gold standard is the albumin creatinine ratio. Diabetes status, serum creatinine and cholesterol level could not be verified as not all the study participants on the second visit gave their blood samples. Of 6505 eligible participants, 2805 had their UAC values in the first and second visits. Although missing values were analysed separately and did not show any systematic bias based on age, smoking and glycaemic status, the data were dominated by male participants aged 44–76. Over 90% of the study participants were of white European ethnicity. Smoking prevalence in the UK Biobank participants was 10.5%, while the Office for National Statistics (ONS) showed smoking prevalence in the UK is 13.3% in 2021.^79^ Only one-third of the UK Biobank study participants were from the least deprived background, which explains the lower rate of smoking, as the ONS data suggest smoking prevalence in the deprived areas is four times more than in the affluent areas.^80^ Therefore, findings from this study cannot be generalised.

People with pre-existing kidney disease and abnormal UAC values were not excluded. This is a limitation of the study as people with kidney disease are likely to have progression of albuminuria irrespective of smoking status.

## supplementary material

10.1136/bmjph-2023-000172 online supplemental file 1

## Data Availability

Data are available on reasonable request.
