# Supplementary material for: Relationship of cardiorenal risk factors with albuminuria based on age, smoking, glycaemic status and BMI: a retrospective cohort study of the UK Biobank data
Source: BMJ Public Health. 2023 Nov 24;1(1):e000172. doi: 10.1136/bmjph-2023-000172 (PMC11812708; doi:10.1136/bmjph-2023-000172)
Supplement: online supplemental file 1 [file bmjph-1-1-s001.pdf]

Supplementary material

A. *Descriptive statistics – participants who had UAC value in the second visit.*

Statistics

		Age_2nd_visit	Age2_Age1	Waist circumference_2	Age_started_smoking_exsmokers	Age_started_smoking_current_smokers	Age_stopped_smoking_2	Duration_of_smoking_exsmokers	Duration_of_smoking_current_smokers	Years_of_abstinence	Age_diabetes_diagnosed	Age_hypertension_diagnosed	SBP_2	DBP_2	BMI_2	UAC_2	Creatinine2_Creatinene	HbA1c_2	Cholesterol_2
N	Valid	6505	6505	6497	1695	369	633	1695	60	633	386	1979	6081	6081	6485	6505	5271	4560	5712
	Missing	0	0	8	4810	6136	5872	4810	6445	5872	6119	4526	424	424	20	0	1234	1945	793
Mean		62.82	4.4008	93.108	17.20	18.39	39.79	22.08	39.22	23.4218	52.16	47.03	138.97	83.03	27.726374	27.650	3.1049	37.38	5.60
Std. Deviation		7.478	1.01648	13.8346	3.672	7.199	12.680	12.130	9.361	13.46399	12.227	16.365	18.759	10.220	4.9660296	103.0703	13.24281	6.963	1.221

Sex

		Frequency	Percent	Valid Percent	Cumulative Percent
Valid	0	2999	46.1	46.1	46.1
	1	3506	53.9	53.9	100.0
	Total	6505	100.0	100.0	

Change_in_UAC

		Frequency	Percent	Valid Percent	Cumulative Percent
Valid	Regressor	987	15.2	35.2	35.2
	No Change	312	4.8	11.1	46.3
	Progressor	1506	23.2	53.7	100.0
	Total	2805	43.1	100.0	
Missing	System	3700	56.9		
Total		6505	100.0		

B. Non-parametric test to determine the distribution of data

Hypothesis Test Summary

	Null Hypothesis	Test	Sig.	Decision
1	The distribution of UAC_2 is the same across categories of progressor_regressor.	Independent-Samples Mann-Whitney U Test	.000	Reject the null hypothesis.
2	The distribution of SBP_2 is the same across categories of progressor_regressor.	Independent-Samples Mann-Whitney U Test	.827	Retain the null hypothesis.
3	The distribution of Waist circumference_2 is the same across categories of progressor_regressor.	Independent-Samples Mann-Whitney U Test	.532	Retain the null hypothesis.
4	The distribution of BMI_2 is the same across categories of progressor_regressor.	Independent-Samples Mann-Whitney U Test	.904	Retain the null hypothesis.
5	The distribution of Cholesterol_2 is the same across categories of progressor_regressor.	Independent-Samples Mann-Whitney U Test	.250	Retain the null hypothesis.
6	The distribution of Creatinine_2 is the same across categories of progressor_regressor.	Independent-Samples Mann-Whitney U Test	.200	Retain the null hypothesis.
7	The distribution of HbA1c_2 is the same across categories of progressor_regressor.	Independent-Samples Mann-Whitney U Test	.095	Retain the null hypothesis.
8	The distribution of Age_started_smoking_exsmokers is the same across categories of progressor_regressor.	Independent-Samples Mann-Whitney U Test	.306	Retain the null hypothesis.
9	The distribution of Age_stopped_smoking is the same across categories of progressor_regressor.	Independent-Samples Mann-Whitney U Test	.342	Retain the null hypothesis.
10	The distribution of Years_of_abstinence is the same across categories of progressor_regressor.	Independent-Samples Mann-Whitney U Test	.610	Retain the null hypothesis.
11	The distribution of Age_diabetes_diagnosed is the same across categories of progressor_regressor.	Independent-Samples Mann-Whitney U Test	.193	Retain the null hypothesis.
12	The distribution of Age_hypertension_diagnosed is the same across categories of progressor_regressor.	Independent-Samples Mann-Whitney U Test	.836	Retain the null hypothesis.
13	The distribution of Age_2nd_visit is the same across categories of progressor_regressor.	Independent-Samples Mann-Whitney U Test	.003	Reject the null hypothesis.

Asymptotic significances are displayed. The significance level is .050.

C.

Independent Samples Test

		Levene's Test for Equality of Variances		t-test for Equality of Means						
									95% Confidence Interval of the Difference	
		F	Sig.	t	df	Sig. (2-tailed)	Mean Difference	Std. Error Difference	Lower	Upper
Age_2nd_visit	Equal variances assumed	4.131	.042	-3.025	2491	.003	-.914	.302	-1.506	-.321
	Equal variances not assumed			-3.004	2059.180	.003	-.914	.304	-1.510	-.317
UAC_2	Equal variances assumed	45.261	.000	-5.756	2491	.000	-36.5848	6.3554	-49.0472	-24.1223
	Equal variances not assumed			-6.912	1812.294	.000	-36.5848	5.2926	-46.9650	-26.2045
DBP_2	Equal variances assumed	.704	.402	2.727	2365	.006	1.209	.443	.340	2.079
	Equal variances not assumed			2.713	1976.549	.007	1.209	.446	.335	2.084
SBP_2	Equal variances assumed	.002	.961	.503	2365	.615	.412	.820	-1.195	2.020
	Equal variances not assumed			.501	1984.020	.616	.412	.823	-1.202	2.026
BMI_2	Equal variances assumed	.119	.730	.355	2480	.723	.0763619	.2153150	-.3458539	.4985777
	Equal variances not assumed			.356	2120.517	.722	.0763619	.2147786	-.3448368	.4975605
Waist circumference_2	Equal variances assumed	.601	.438	-.550	2489	.582	-.3198	.5816	-1.4603	.8206
	Equal variances not assumed			-.553	2145.055	.580	-.3198	.5782	-1.4537	.8141
Creatinine_2	Equal variances assumed	3.920	.048	-2.051	2197	.040	-1.860	.907	-3.639	-.082
	Equal variances not assumed			-2.180	2151.115	.029	-1.860	.853	-3.533	-.187
HbA1c_2	Equal variances assumed	.545	.460	-.515	1752	.607	-.199	.386	-.957	.559
	Equal variances not assumed			-.514	1511.697	.607	-.199	.387	-.959	.561

D. Descriptive statistics of ex-smokers

Statistics

		Duration_of_smoking_exsmokers	Duration of abstinence	Age_started_smoking_exsmokers	Age_2nd_visit	Age_stopped_smoking	DBP_2	SBP_2	Waist circumference_2	BMI_2	Cholestero 2
N	Valid	532	532	532	532	532	500	500	532	531	4
	Missing	0	0	0	0	0	32	32	0	1	
Mean		21.33	24.88	17.18	63.39	38.51	83.51	140.43	96.015	28.516186	5.508
Std. Deviation		11.327	11.690	3.342	6.937	11.136	10.270	20.246	13.4375	4.9860518	1.2048

Sex

		Frequency	Percent	Valid Percent	Cumulative Percent
Valid	0	188	35.3	35.3	35.3
	1	344	64.7	64.7	100.0
	Total	532	100.0	100.0	

E. Chi-squared test to show significance of the relationship between the progression groups with glycaemic and smoking status.

Crosstab

Count

		new_smoking_status2			Total
		Non smoker	Ex-smoker	Current smoker	
Change_in_UAC	Regressor	911	57	18	986
	No Change	299	8	5	312
	Progressor	1397	81	26	1504
Total		2607	146	49	2802

Crosstab

Count

		Diabetes_status_2			Total
		non diabetic	prediabetic	diabetic	
Change_in_UAC	Regressor	579	65	66	710
	No Change	201	12	10	223
	Progressor	850	99	95	1044
Total		1630	176	171	1977

Progression and smoking status

Chi-Square Tests

	Value	df	Asymptotic Significance (2-sided)
Pearson Chi-Square	5.282[a]	4	.260
Likelihood Ratio	6.233	4	.182
Linear-by-Linear Association	.095	1	.758
N of Valid Cases	2802		

a. 0 cells (0.0%) have expected count less than 5. The minimum expected count is 5.46.

Progression and diabetes status

Chi-Square Tests

	Value	df	Asymptotic Significance (2-sided)
Pearson Chi-Square	10.414[a]	4	.034
Likelihood Ratio	11.829	4	.019
Linear-by-Linear Association	.032	1	.857
N of Valid Cases	1977		

a. 0 cells (0.0%) have expected count less than 5. The minimum expected count is 19.29.

F. *Pearson's correlation*

Correlations

			UAC_2	Age_2nd_visit	SBP_2	DBP_2	Waist circumference_2	BMI_2	Cholesterol_2	Creatinine_2	HbA1c_2	HDL_2	LDL_2	Duration_of_smoking_current_smokers	Duration_of_smoking_exsmokers
Spearman's rho	UAC_2	Correlation Coefficient	1.000	.088**	.071**	.037**	.102**	.081**	-.078**	.093**	.102**	-.061**	-.077**	.364**	.083**
		Sig. (2-tailed)	.	.000	.000	.004	.000	.000	.000	.000	.000	.000	.000	.004	.001
		N	6505	6505	6081	6081	6497	6485	5712	5698	4560	5012	5696	60	1695
	Age_2nd_visit	Correlation Coefficient	.088**	1.000	.272**	-.031*	.071**	.004	-.119**	.115**	.213**	.014	-.129**	.879**	.194**
		Sig. (2-tailed)	.000	.	.000	.015	.000	.767	.000	.000	.000	.337	.000	.000	.000
		N	6505	6505	6081	6081	6497	6485	5712	5698	4560	5012	5696	60	1695
	SBP_2	Correlation Coefficient	.071**	.272**	1.000	.657**	.141**	.112**	-.024	.111**	.081**	-.039**	-.021	.242	.047
		Sig. (2-tailed)	.000	.000	.	.000	.000	.000	.078	.000	.000	.008	.124	.070	.062
		N	6081	6081	6081	6081	6074	6063	5340	5326	4233	4680	5325	57	1575
	DBP_2	Correlation Coefficient	.037**	-.031*	.657**	1.000	.234**	.218**	.006	.121**	.008	-.102**	.030*	-.074	.022
		Sig. (2-tailed)	.004	.015	.000	.	.000	.000	.662	.000	.613	.000	.027	.585	.387
		N	6081	6081	6081	6081	6074	6063	5340	5326	4233	4680	5325	57	1575
	Waist circumference_2	Correlation Coefficient	.102**	.071**	.141**	.234**	1.000	.815**	-.238**	.327**	.231**	-.491**	-.149**	.279*	.163**
		Sig. (2-tailed)	.000	.000	.000	.000	.	.000	.000	.000	.000	.000	.000	.031	.000
		N	6497	6497	6074	6074	6497	6482	5705	5691	4555	5007	5689	60	1694
	BMI_2	Correlation Coefficient	.081**	.004	.112**	.218**	.815**	1.000	-.145**	.157**	.252**	-.383**	-.075**	.170	.130**
		Sig. (2-tailed)	.000	.767	.000	.000	.000	.	.000	.000	.000	.000	.000	.194	.000
		N	6485	6485	6063	6063	6482	6485	5697	5683	4546	4999	5681	60	1689
	Cholesterol_2	Correlation Coefficient	-.078**	-.119**	-.024	.006	-.238**	-.145**	1.000	-.209**	-.160**	.437**	.952**	-.182	-.128**
		Sig. (2-tailed)	.000	.000	.078	.662	.000	.000	.	.000	.000	.000	.000	.205	.000
		N	5712	5712	5340	5340	5705	5697	5712	5698	4064	5012	5696	50	1485
	Creatinine_2	Correlation Coefficient	.093**	.115**	.111**	.121**	.327**	.157**	-.209**	1.000	.051**	-.316**	-.145**	.027	.099**
		Sig. (2-tailed)	.000	.000	.000	.000	.000	.000	.000	.	.001	.000	.000	.853	.000
		N	5698	5698	5326	5326	5691	5683	5698	5698	4054	5001	5687	50	1480
	HbA1c_2	Correlation Coefficient	.102**	.213**	.081**	.008	.231**	.252**	-.160**	.051**	1.000	-.151**	-.149**	.026	.180**
		Sig. (2-tailed)	.000	.000	.000	.613	.000	.000	.000	.001	.	.000	.000	.880	.000
		N	4560	4560	4233	4233	4555	4546	4064	4054	4560	3603	4054	36	1184
	HDL_2	Correlation Coefficient	-.061**	.014	-.039**	-.102**	-.491**	-.383**	.437**	-.316**	-.151**	1.000	.230**	-.024	-.085**
		Sig. (2-tailed)	.000	.337	.008	.000	.000	.000	.000	.000	.000	.	.000	.878	.002
		N	5012	5012	4680	4680	5007	4999	5012	5001	3603	5012	5000	43	1302
	LDL_2	Correlation Coefficient	-.077**	-.129**	-.021	.030*	-.149**	-.075**	.952**	-.145**	-.149**	.230**	1.000	-.218	-.123**
		Sig. (2-tailed)	.000	.000	.124	.027	.000	.000	.000	.000	.000	.000	.	.128	.000
		N	5696	5696	5325	5325	5689	5681	5696	5687	4054	5000	5696	50	1483
	Duration_of_smoking_current_smokers	Correlation Coefficient	.364**	.879**	.242	-.074	.279*	.170	-.182	.027	.026	-.024	-.218	1.000	.526**
		Sig. (2-tailed)	.004	.000	.070	.585	.031	.194	.205	.853	.880	.878	.128	.	.000
		N	60	60	57	57	60	60	50	50	36	43	50	60	60
	Duration_of_smoking_exsmokers	Correlation Coefficient	.083**	.194**	.047	.022	.163**	.130**	-.128**	.099**	.180**	-.085**	-.123**	.526**	1.000
		Sig. (2-tailed)	.001	.000	.062	.387	.000	.000	.000	.000	.000	.002	.000	.000	.
		N	1695	1695	1575	1575	1694	1689	1485	1480	1184	1302	1483	60	1695

**. Correlation is significant at the 0.01 level (2-tailed).

*. Correlation is significant at the 0.05 level (2-tailed).

G. Missing data analyses

Sex

		Frequency	Percent	Valid Percent	Cumulative Percent
Valid	0	1876	50.7	50.7	50.7
	1	1824	49.3	49.3	100.0
	Total	3700	100.0	100.0	

Age_groups

		Frequency	Percent	Valid Percent	Cumulative Percent
Valid	44-54	657	17.8	17.8	17.8
	55-64	1313	35.5	35.5	53.2
	65-70	1211	32.7	32.7	86.0
	>70	519	14.0	14.0	100.0
	Total	3700	100.0	100.0	

Diabetes_status_2

		Frequency	Percent	Valid Percent	Cumulative Percent
Valid	non diabetic	2324	62.8	90.0	90.0
	prediabetic	142	3.8	5.5	95.5
	diabetic	117	3.2	4.5	100.0
	Total	2583	69.8	100.0	
Missing	System	1117	30.2		
Total		3700	100.0		

H. Analyses of people with no change between the two visits

Sex

		Frequency	Percent	Valid Percent	Cumulative Percent
Valid	0	144	46.2	46.2	46.2
	1	168	53.8	53.8	100.0
	Total	312	100.0	100.0	

Age_groups

		Frequency	Percent	Valid Percent	Cumulative Percent
Valid	44-54	61	19.6	19.6	19.6
	55-64	118	37.8	37.8	57.4
	65-70	94	30.1	30.1	87.5
	>70	39	12.5	12.5	100.0
	Total	312	100.0	100.0	

Diabetes_status_2

		Frequency	Percent	Valid Percent	Cumulative Percent
Valid	non diabetic	201	64.4	90.1	90.1
	prediabetic	12	3.8	5.4	95.5
	diabetic	10	3.2	4.5	100.0
	Total	223	71.5	100.0	
Missing	System	89	28.5		
Total		312	100.0		

new_smoking_status2

		Frequency	Percent	Valid Percent	Cumulative Percent
Valid	Non smoker	299	95.8	95.8	95.8
	Ex-smoker	8	2.6	2.6	98.4
	Current smoker	5	1.6	1.6	100.0
	Total	312	100.0	100.0	

I. Model 1 – Predictors of progression: visit 1 and visit 2.

Variables in the Equation

		B	S.E.	Wald	df	Sig.	Exp(B)	95% C.I.for EXP(B) Lower	Upper
Step 1[a]	Sex(1)	.198	.133	2.225	1	.136	1.219	.940	1.581
	Age2_Age1	.180	.064	7.922	1	.005	1.198	1.056	1.358
	BMI2_BMI1	.026	.037	.501	1	.479	1.026	.955	1.102
	HbA1c2_HbA1c1	.028	.012	5.068	1	.024	1.028	1.004	1.053
	SBP2_SBP1	.001	.007	.033	1	.857	1.001	.988	1.014
	Cholesterol2_Cholesterol1	.082	.065	1.555	1	.212	1.085	.954	1.234
	Creatinine2_Creatinene	-.008	.006	2.029	1	.154	.992	.980	1.003
	HDL2_HDL1	-.640	.330	3.767	1	.052	.527	.276	1.006
	new_smoking_status2			.901	2	.637			
	new_smoking_status2(1)	.197	.291	.461	1	.497	1.218	.689	2.153
	new_smoking_status2(2)	-.330	.514	.411	1	.521	.719	.263	1.969
	Constant	-.468	.302	2.404	1	.121	.626		

a. Variable(s) entered on step 1: Sex, Age2_Age1, BMI2_BMI1, HbA1c2_HbA1c1, SBP2_SBP1, Cholesterol2_Cholesterol1, Creatinine2_Creatinene, HDL2_HDL1, new_smoking_status2.

J. Model 2a – Predictors of albuminuria in ex-smokers: with waist circumference and the duration of smoking

Variables in the Equation

		B	S.E.	Wald	df	Sig.	Exp(B)	95% C.I.for EXP(B) Lower	Upper
Step 1[a]	Sex(1)	-.286	.532	.287	1	.592	.752	.265	2.134
	Age_2nd_visit	.036	.031	1.304	1	.254	1.037	.975	1.102
	Duration_of_smoking_exsmokers	.042	.021	4.010	1	.045	1.043	1.001	1.086
	Cholesterol_2	.015	.192	.006	1	.936	1.015	.698	1.478
	HbA1c2_HbA1c1	-.020	.038	.287	1	.592	.980	.910	1.055
	Waist circumference_2	-.004	.018	.061	1	.806	.996	.962	1.031
	DBP_2	-.039	.022	3.225	1	.073	.962	.921	1.004
	Constant	1.075	3.336	.104	1	.747	2.930		

a. Variable(s) entered on step 1: Sex, Age_2nd_visit, Duration_of_smoking_exsmokers, Cholesterol_2, HbA1c2_HbA1c1, Waist circumference_2, DBP_2.

K. *Model 2b - Predictors of albuminuria in ex-smokers: with BMI and the duration of smoking*

Variables in the Equation

		B	S.E.	Wald	df	Sig.	Exp(B)	95% C.I.for EXP(B) Lower	Upper
Step 1[a]	Sex(1)	-.345	.504	.470	1	.493	.708	.264	1.901
	Age_2nd_visit	.043	.032	1.740	1	.187	1.044	.979	1.112
	Duration_of_smoking_ex smokers	.037	.021	3.299	1	.069	1.038	.997	1.081
	Cholesterol_2	.038	.188	.041	1	.840	1.039	.719	1.500
	HbA1c2_HbA1c1	-.027	.038	.491	1	.483	.973	.903	1.049
	DBP_2	-.040	.022	3.395	1	.065	.960	.920	1.003
	BMI_2	.029	.046	.406	1	.524	1.030	.941	1.128
	Constant	-.527	3.234	.026	1	.871	.591		

a. Variable(s) entered on step 1: Sex, Age_2nd_visit, Duration_of_smoking_exsmokers, Cholesterol_2, HbA1c2_HbA1c1, DBP_2, BMI_2.

L. *Model 2c - Predictors of progression in ex-smokers: without BMI & waist circumference and the duration of smoking*

Variables in the Equation

		B	S.E.	Wald	df	Sig.	Exp(B)	95% C.I.for EXP(B) Lower	Upper
Step 1[a]	Sex(1)	-.326	.505	.417	1	.518	.722	.268	1.942
	Age_2nd_visit	.037	.031	1.426	1	.232	1.038	.977	1.103
	HbA1c2_HbA1c1	-.022	.037	.344	1	.558	.978	.910	1.052
	DBP_2	-.039	.022	3.264	1	.071	.961	.921	1.003
	Cholesterol_2	.025	.187	.017	1	.895	1.025	.710	1.479
	Duration_of_smoking_ex smokers	.040	.020	4.067	1	.044	1.041	1.001	1.083
	Constant	.599	2.713	.049	1	.825	1.820		

a. Variable(s) entered on step 1: Sex, Age_2nd_visit, HbA1c2_HbA1c1, DBP_2, Cholesterol_2, Duration_of_smoking_exsmokers.

M. *Model 3a - Predictors of progression in ex-smokers: with waist circumference and the duration of abstinence*

Variables in the Equation

		B	S.E.	Wald	df	Sig.	Exp(B)	95% C.I.for EXP(B)	
								Lower	Upper
Step 1[a]	Sex(1)	-.228	.540	.178	1	.673	.796	.276	2.296
	Age_2nd_visit	.085	.033	6.495	1	.011	1.089	1.020	1.162
	HbA1c2_HbA1c1	-.015	.038	.157	1	.692	.985	.914	1.061
	DBP_2	-.039	.022	3.103	1	.078	.962	.921	1.004
	Cholesterol_2	.005	.195	.001	1	.978	1.005	.687	1.472
	New_Duration_of_abstinence	-.056	.022	6.428	1	.011	.945	.905	.987
	Waist circumference_2	-.009	.018	.266	1	.606	.991	.956	1.027
	Constant	.708	3.310	.046	1	.831	2.030		

a. Variable(s) entered on step 1: Sex, Age_2nd_visit, HbA1c2_HbA1c1, DBP_2, Cholesterol_2, New_Duration_of_abstinence, Waist circumference_2.

N. *Model 3b - Predictors of progression in ex-smokers: with BMI and the duration of abstinence*

Variables in the Equation

		B	S.E.	Wald	df	Sig.	Exp(B)	95% C.I.for EXP(B)	
								Lower	Upper
Step 1[a]	Sex(1)	-.331	.510	.420	1	.517	.719	.264	1.952
	Age_2nd_visit	.085	.033	6.545	1	.011	1.089	1.020	1.163
	HbA1c2_HbA1c1	-.022	.039	.334	1	.563	.978	.906	1.055
	DBP_2	-.040	.022	3.279	1	.070	.961	.920	1.003
	Cholesterol_2	.033	.190	.030	1	.862	1.034	.712	1.501
	New_Duration_of_abstinence	-.050	.022	5.372	1	.020	.951	.911	.992
	BMI_2	.019	.047	.157	1	.692	1.019	.929	1.117
	Constant	-.911	3.207	.081	1	.776	.402		

a. Variable(s) entered on step 1: Sex, Age_2nd_visit, HbA1c2_HbA1c1, DBP_2, Cholesterol_2, New_Duration_of_abstinence, BMI_2.

O. Model 3c - Predictors of progression in ex-smokers: without BMI and waist circumference and the duration of abstinence

Variables in the Equation

		B	S.E.	Wald	df	Sig.	Exp(B)	95% C.I.for EXP(B)	
								Lower	Upper
Step 1[a]	Sex(1)	-.317	.510	.387	1	.534	.728	.268	1.978
	Age_2nd_visit	.084	.033	6.429	1	.011	1.088	1.019	1.161
	HbA1c2_HbA1c1	-.019	.038	.253	1	.615	.981	.912	1.056
	DBP_2	-.039	.022	3.196	1	.074	.961	.921	1.004
	Cholesterol_2	.025	.190	.018	1	.894	1.026	.707	1.487
	New_Duration_of_abstinence	-.053	.021	6.350	1	.012	.949	.911	.988
	Constant	-.248	2.736	.008	1	.928	.780		

a. Variable(s) entered on step 1: Sex, Age_2nd_visit, HbA1c2_HbA1c1, DBP_2, Cholesterol_2, New_Duration_of_abstinence.
